# Gene expression profiling of the anti-glioma effect of Cilengitide

**DOI:** 10.1186/2193-1801-2-160

**Published:** 2013-04-15

**Authors:** Manabu Onishi, Kazuhiko Kurozumi, Tomotsugu Ichikawa, Hiroyuki Michiue, Kentaro Fujii, Joji Ishida, Yosuke Shimazu, E Antonio Chiocca, Balveen Kaur, Isao Date

**Affiliations:** 1 Department of Neurological Surgery, Okayama University Graduate School of Medicine, Dentistry and Pharmaceutical Sciences, 2-5-1, Shikata-cho, Kita-ku, Okayama, 700-8558 Japan; 2 Department of Physiology, Okayama University Graduate School of Medicine, Dentistry and Pharmaceutical Sciences, Okayama, Japan; 3 Brigham and Women’s Hospital, Neurosurgery, Boston, MA USA; 4 Department of Neurological Surgery, Dardinger Laboratory for Neuro-oncology and Neurosciences, The Ohio State University, Columbus, OH USA

**Keywords:** Glioma, Integrin, Cilengitide, Gene expression profiling, Apoptosis

## Abstract

Cilengitide (EMD121974), an inhibitor of the adhesive function of integrins, demonstrated preclinical efficacy against malignant glioma. It is speculated that cilengitide can inhibit tumor growth, invasion, and angiogenesis. However, the effects of cilengitide on these processes have not been sufficiently examined. In this study, we investigated the anti-glioma effect of cilengitide using DNA microarray analysis. U87ΔEGFR cells (human malignant glioma cell line) were used for this experiment. The cells were harvested after 16 h of cilengitide treatment, and mRNA was extracted. Gene expression and pathway analyses were performed using a DNA microarray (CodeLink™Human Whole Genome Bioarray). The expression of 265 genes was changed with cilengitide treatment. The expression of 214 genes was up-regulated by more than 4-fold and the expression of 51 genes was down-regulated by more than 4-fold compared to the controls. In pathway analysis, “apoptotic cleavage of cellular proteins” and “TNF receptor signaling pathway” were over-represented. Apoptotic-associated genes such as caspase 8 were up-regulated. Gene expression profiling revealed more detailed mechanism of the anti-glioma effect of cilengitide. Genes associated with apoptosis were over-represented following cilengitide treatment.

## Introduction

Gliomas are the most frequent primary intracranial neoplasm in adults and are invariably fatal. The median survival of aggressively treated patients with glioblastoma is approximately 14.6 months (Stupp & Weber 2005). The resistance of gliomas to the conventional therapeutic regimen of surgery, radiotherapy, and chemotherapy has prompted many investigators to seek novel therapeutic approaches for this fatal disease (Chatterjee et al. 2000). Moreover, alterations of the epidermal growth factor receptor (*EGFR*) gene are common in some forms of cancer and the most frequent is a deletion of exons 2–7. It was previously reported that this mutant receptor, called ΔEGFR, confers enhanced tumorigenicity on glioblastoma cells through elevated proliferation and reduced apoptotic rates in vivo (Narita et al. 2002).

Integrins control the attachment of cells to the extracellular matrix (ECM) and participate in cellular defense against genotoxic assaults (Hynes 2002). Integrins are expressed in tumor cells and tumor endothelial cells (Varner & Cheresh 1996a; Varner & Cheresh 1996b; Varner et al. 1995), and they play important roles in angiogenesis and invasion in glioma (Friedlander et al. 1995; Brooks et al. 1994a; Brooks et al. 1994b). αvβ3 and αvβ5 integrins regulate cell adhesion (Hodivala-Dilke et al. 2003; Leavesley et al. 1992), and inhibitors of these integrins suppress tumor growth in certain pre-clinical models (MacDonald et al. 2001). Therefore, integrins have attracted attention as potential therapeutic targets in glioma.

Currently, αvβ3 and αvβ5 integrin antagonists including cilengitide (EMD121974), which is a cyclic RGD-containing peptide (Xiong et al. 2001), are in clinical trials. This drug is reportedly able to penetrate the blood brain barrier (BBB) in vivo (Nabors et al. 2007). Cilengitide induces anoikis in angiogenic blood vessels and brain tumor cells in vitro (Oliveira-Ferrer et al. 2008; Alghisi et al. 2009); however, the mechanisms underlying its cytotoxic effects are not completely understood.

There have been no microarray studies to date that examined the changes in gene expression after cilengitide treatment. U87ΔEGFR cells were previously shown to have increased tumorigenicity via elevated proliferation and reduced apoptosis compared to U87 cells, which cause a more aggressive phenotype than that of the parental cell line (Narita et al. 2002). In this study, we profiled and examined the gene expression of over 57000 genes using the most comprehensive GeneChip microarrays available (CodeLink™ Human Whole Genome Bioarray), and identified differentially expressed genes between untreated glioma cells (U87ΔEGFR) and cilengitide-treated glioma cells to reveal more detail mechanism of anti-glioma effect of cilengitide.

## Materials and methods

### Glioma cell line and drug

Glioma cell lines, U87ΔEGFR, were seeded in tissue culture dishes (BD Falcon, Franklin Lakes, NJ, USA) and cultured in Dulbecco’s Modified Eagle’s Medium (DMEM) supplemented with 10% fetal bovine serum (FBS), 100 U penicillin, and 0.1 mg/mL streptomycin. U87MG, Gli36Δ5, and U251 human glioma cells were also prepared and maintained as described previously (Kambara et al. 2005). Cilengitide was generously provided by Merck KgaA and the National Cancer Institute, National Institutes of Health.

### Cell surface immunofluorescence assay

U87**Δ**EGFR cells were seeded on 4 Chamber Polystyrene Vessel Tissue Culture Treated Glass Slides (BD Falcon, Franklin Lakes, NJ, USA) and incubated overnight. For immunofluorescence, the cells were fixed in 4% paraformaldehyde in phosphate-buffered saline (PBS) for 15 min. After the cells were fixed, they were rinsed 3 times with PBS. Nonspecific binding was blocked by incubation in a blocking buffer containing 2% bovine serum albumin in PBS for 30 min at room temperature. The cells were incubated overnight at 4°C with a mouse anti-human integrin αvβ3 monoclonal antibody (Millipore Corporation, Billerica, MA, USA) or mouse anti-human integrin αvβ5 monoclonal antibody (Abcam, Cambridge, UK) diluted 1:100 in blocking buffer. The cells were washed 3 times in blocking buffer for 5 min before incubation with a secondary anti-mouse CY3-conjugated antibody (Jackson ImmunoResearch Laboratories, Inc., West Grove, PA, USA) diluted 1:300 in blocking buffer for 2 h at room temperature in the dark. After 3 washes in PBS, the cells were counterstained with 4^′^, 6-diamino-2-phenylindole (DAPI; 1:500) (Invitrogen, Carlsbad, CA, USA) (100 ng/mL) for 20 min at room temperature. The slides were washed 3 times in PBS and mounted.

### Water-soluble tetrazolium-1 assay

A water-soluble tetrazolium (WST)-1 assay (Roche Diagnostics) was performed according to the manufacturer’s instructions. Briefly, cells treated with saline (control cells) or Cilengitide (1.0 μM) (Cilengitide was generously provided by Merck KGaA and the National Cancer Institute, NIH) were plated in a 96-well plate at a concentration of 25,000 cells/mL. Cell survival was measured at the indicated time points by adding 10 μL of a 1:3 (v/v) diluted ready-to-use WST-1 cell proliferation reagent stock solution (Roche, Mannheim). The samples were incubated for 60–240 min and absorption was measured with an MTP-120 micro plate reader (CORONA ELECTRIC Co., Ibaragi, Japan) at 450 nm wavelength using a 620 nm reference filter. After subtraction of the background absorption, the mean value of the untreated control cells was set as 100%.

### Microarray analysis

U87ΔEGFR cells treated with cilengitide (1.0 μM for 16 h) and untreated control U87ΔEGFR cells were analyzed using a CodeLink™ Human Whole Genome Bioarray (Applied Microarrays, Inc., Tempe, AZ, USA). We entrusted the microarray analyses to Filgen, Inc. (Nagoya, Japan). Briefly, for each bioarray, 10 μg of cRNA in a 25 μL total volume were added to 5 μL of 5× fragmentation buffer, which was then incubated at 94°C for 20 min. Thereafter, 10 μg of fragmented cRNA, 78 μL of hybridization buffer component A, and 130 μL of hybridization buffer component B were added, and the final volume was brought up to 260 μL with water. The resultant hybridization reaction mixture was incubated at 90°C for 5 min, after which 250 μL were slowly injected into the input port of each array, and the ports were sealed with sealing strips. The bioarrays were then incubated for 18 h at 37°C while shaking at 300 rpm. A consistent hybridization time was maintained for comparative experiments. Following the incubation, the bioarrays were washed with 0.75 TNT buffer (0.10 M Tris–HCl, pH 7.6, 0.15 M NaCl, 0.05% Tween 20) and incubated at 46°C for 1 h. Each slot of the small reagent reservoir was then filled with 3.4 mL of Cy5-Streptavidin working solution, and the array was incubated at 25°C for another 30 min. Thereafter, the bioarrays were washed 4 times for 5 min each with 1 × TNT buffer at 25°C, rinsed twice in 0.1× SSC (Ambion, Austin, TX, USA)/0.05% Tween 20 for 30 s each, and immediately dried by centrifugation for 3 min at 25°C. Finally, the arrays were scanned using a GenePix4000B Array Scanner (Molecular Devices, Sunnyvale, CA, USA). A gene was defined as being upregulated when the cilengitide treatment/control average intensity ratio was >4.0, and downregulated when the cilengitide treatment/control ratio was <0.25. We performed pathway analysis on the genes that expressed increase and decrease using Microarray Data Analysis Tool Ver3.2 (Filgen, Inc). The data were extracted using the following criteria: Z-score > 0 and P-value < 0.05 (Ichii et al. 2011; Yoshino et al. 2011).

### Quantitative reverse-transcription polymerase chain reaction (QRT-PCR)

Total RNA was isolated from cultured U87ΔEGFR cells treated with cilengitide (1.0 μM for 16 h) and untreated control U87ΔEGFR cells using an RNeasy® Mini Kit (QIAGEN, Hilden, Germany). In vivo, that RNA was extracted from the brain tumor tissue of rat that had been treated with PBS or with Cilengitide with the use of TRIZOL reagent (Invitrogen, Carlsbad, CA, USA), according to the manufacturer’s instructions. Those RNA were reverse transcribed with oligo dT primers using the SuperScript III First-Strand Synthesis System for RT-PCR (Invitrogen, Carlsbad, CA, USA) according to manufacturer’s instructions. Primers specific for each gene were designed using Primer 3 Plus Software (http://www.bioinformatics.nl/cgi-bin/primer3plus/primer3plus.cgi) and synthesized by Invitrogen. The resulting cDNA was amplified by PCR using gene-specific primers and the 7300 Real Time PCR system (Applied Biosystems, Foster City, CA, USA) and QuantiTectTM SYBR® Green PCR Kit (QIAGEN, Hilden, Germany). A log-linear relationship between the amplification curve and quantity of cDNA in the range of 1–1000 copies was observed. The cycle number at the threshold was used as the threshold cycle (Ct). The different expression of mRNA was detected from 2-ΔΔCt using the 7300 Real Time PCR System with Sequence Detection Software (version 1.4; Applied Biosystems, Foster City, CA, USA). The amount of cDNA in each sample was normalized to the crossing point of the housekeeping gene glyceraldehyde 3-phosphate dehydrogenase (GAPDH). The following thermal cycling parameters were used: denaturation at 95°C for 10 min followed by 45 cycles at 94°C for 15 s, 50°C for 30 s, and 72°C for 30 s. The relative mRNA upregulation for each gene in the control was calculated using their respective crossing points with the following formula, as previously described (Kurozumi et al. 2007):

*F = 2*^*(TH − TG) − (OH − OG)*^ where, *F* = fold difference, *T* = control, *O* = treated cell or tumor, *H* = housekeeping (GAPDH), and *G* = gene of interest.

CASPASE 8 primers

CASP8 F (forward), 5-TGCAGGGTCTCACTCTGTTG-3

CASP8 R (reverse), 5-TTGATTTTGGAGGGATCTCG-3

protein kinase C, zeta primers

PKCZ F (forward), 5-GTTATCGATGGGATGGATGG-3

PKCZ R (reverse), 5-GCACCAGCTCTTTCTTCACC-3

GAPDH primers

GAPDH F (forward), 5- GAGTCAACGGATTTGGTCGT-3

GAPDH R (reverse), 5-TTGATTTTGGAGGGATCTCG-3

### Activity assay of caspase-3/7 and -8

Caspase-3/7 and -8 activity levels were measured using CellEvent™ Caspase-3/7 Green Detection Reagent (Invitrogen, Carlsbad, CA, USA) and the colorimetric protease assay kit (MBL, CA, USA), respectively, according to the protocol recommended by the manufacturer. Briefly, cells (5,000 cells per well) were plated in 96-well plates in triplicate and treated with cilengitide. Cilengitide (1.0 μM) was added to the medium after 24 h of incubation. The caspase solution was added at 16 h after adding cilengitide. After incubation with these substrates, the absorbance of each well was measured using a microplate reader.

### TdT-mediated dUTP nick end labeling (TUNEL) assay

U87ΔEGFR cells were seeded in 6-well plates (1.0 × 10^4^ cells/well) and cultured in DMEM supplemented with 10% FBS. Cilengitide (10 μM) was added to the medium after 24 h of incubation. After incubation for 16 h at 37°C, the cells were examined for morphological changes. Apoptotic cells were detected with the In Situ Cell Death Detection Kit (Roche, Basel, Switzerland) according to the manufacturer’s instructions.

### Immunoblot analysis

Using immunoblot analysis, we examined whether treatment with cilengitide induced caspase activation via caspase 8 and caspase 3. Western blotting was carried out at a high stringency, essentially as described previously (Kurozumi et al. 2004; Michiue et al. 2005a; Michiue et al. 2005b). The harvested cells with treated each concentration of drug (0.1, 1.0, 10 μM: 16 hr) or each time course (8, 16, 24 hr: 1 μM) were lysed by a sonicator in a boiled buffer containing 1% SDS. The cell lysate (50 μg) was subjected to SDS-PAGE and transferred to nitrocellulose membranes (Hybond ECL, GE Healthcare UK Ltd, England). The blots were probed with each primary antibody. Specific bands were visualized with an enhanced chemiluminescence detection kit (GE Healthcare UK Ltd, England). The following primary rabbit polyclonal antibodies were used: anti-human caspase 8 (1:500; ab44976, abcam, Inc., UK), anti-human caspase 3 (1:250; ab32125, abcam, Inc., UK), and β-actin (1:1000; Sigma-Aldrich, St. Louis, MO, USA).

### Brain xenografts

All experimental animals were housed and handled in accordance with the guidelines of the Okayama University Animal Research Committee. Before implantation, 85–90% confluent U87ΔEGFR cells were trypsinized, rinsed with DMEM supplemented with 10% FBS, and centrifuged at 100 × *g* for 5 min; the resulting pellet was resuspended in PBS, and the cell concentration was adjusted to 1.0 × 10^5^ cells/μL. U87ΔEGFR cells (5 μL) were injected into athymic rats (F344/N-nu/nu; CLEA Japan, Inc., Tokyo, Japan). The animals were anesthetized and placed in stereotactic frames (Narishige, Tokyo, Japan) with their skulls exposed. Tumor cells were injected with a Hamilton syringe (Hamilton, Reno, NV, USA) into the right frontal lobe (4 mm lateral and 1 mm anterior to the bregma at a depth of 4 mm) and the syringe was slowly withdrawn after 5 min to prevent reflux. The skulls were then cleaned, the holes were sealed with bone wax, and the incision was sutured. Cilengitide or PBS was administered 3 times/week intraperitoneally (1 mg/500 μL PBS), starting on day 5 after tumor cell implantation. To assess the gene expression of caspase 8 with QRT-PCR, athymic rats harboring U87ΔEGFR brain tumors were sacrificed at 18 days after tumor implantation. The tumor-bearing right hemispheres of the brains were excised and processed for RNA. For measurements of tumor cell apoptosis, athymic rats were sacrificed at 18 days after tumor implantation. The brains were removed and fixed in 4% paraformaldehyde for at least 24 h.

### TUNEL staining in vivo

Snap-frozen tissue samples were embedded in optimal cutting temperature solution (Sakura Finetek Inc., Torrance, CA, USA) for cryosectioning, and 16-μm cryostat sections were cut. Apoptotic tumor cells were detected using the In Situ Cell Death Detection Kit (Roche, Basel, Switzerland) according to the manufacturer’s instructions.

### Statistical analysis

Student’s *t* test was used to test for statistical significance. Data are presented as the mean ± standard error. All statistical analyses were performed with the use of SPSS statistical software (version 14.0; SPSS, Inc., Chicago, IL, USA).

## Results

### Immunohistochemical analysis of αvβ3 and αvβ5 integrins expression in U87ΔEGFR cells

**Figure 1:**
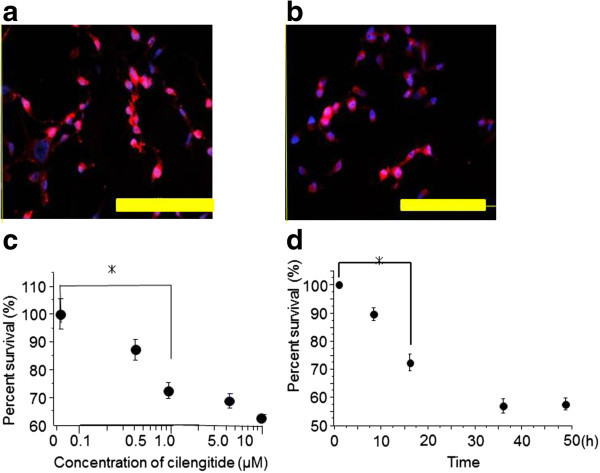
**Immunohistochemical analysis of the αvβ3 and αvβ5 integrins, and WST-1 proliferation/viability assay.** Cultured U87**Δ**EGFR cells were immunopositive for αvβ3 (**a**) and αvβ5 (**b**) integrins (Scale bar = 100 μm). Cilengitide reduced the number of viable cells in a dose and time -dependent manner (*P < 0.05) (mean ± SE) (**c, d**).

Immunofluorescence assays were conducted to determine the expression of αvβ3 and αvβ5 integrins in U87ΔEGFR cells. Cultured U87ΔEGFR cells were immunopositive for αvβ3 and αvβ5 integrins (Figure 1a, b).

### Cytotoxic effects of cilengitide on the U87ΔEGFR glioma cell line in vitro

The direct effects of cilengitide were investigated on glioma cells in vitro. U87ΔEGFR cells were incubated with cilengitide at concentrations of 0–10 μM; 16 h later, the cells were subjected to the WST-1 proliferation/viability assay. Cell viability after 16 h of incubation was decreased in cell cultures treated with cilengitide, reaching statistical significance at 1 μM or higher (Figure 1c). In addition, cell viability was decreased in cell cultures treated with 1 μM cilengitide, reaching statistical significance at 16 h or later (Figure 1d). These cells became sensitive to cilengitide in a concentration- and time-dependent manner.

### Microarray analysis

**Figure 2:**
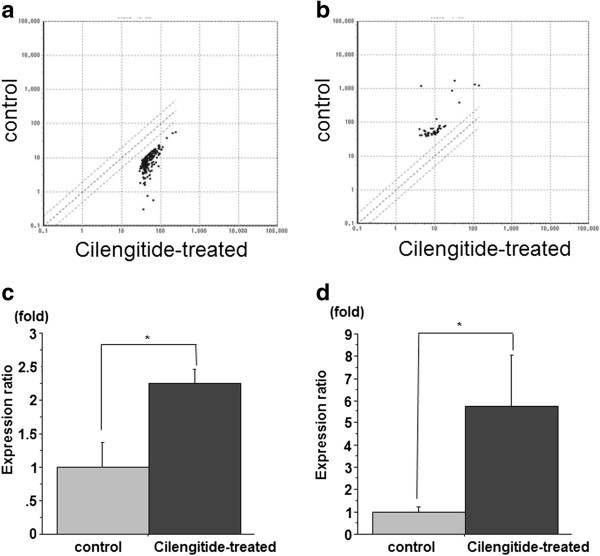
**Microarray and QRT-PCR analyses of cilengitide-treated cells.** There were 265 differentially expressed genes between cilengitide-treated U87ΔEGFR cells and untreated control U87ΔEGFR cells with 214 upregulated (**a**) and 51 downregulated (**b**) genes. Caspase-8 (**c**) and protein kinase C, zeta (**d**) were significantly increased by cilengitide treatment (*P < 0.05) (mean ± SE, n = 3).

Our cell viability assay showed that the decrease in the number of viable cells treated with 1 μM cilengitide reached statistical significance at 16 h. At that time point, differential gene expression was compared between cilengitide-treated U87ΔEGFR cells and untreated control U87ΔEGFR cells (>4 fold change, <0.25 fold change) (Figure 2a, b). There were 265 differentially expressed genes between cilengitide-treated U87ΔEGFR cells and control U87ΔEGFR cells with 214 upregulated and 51 downregulated. We further characterized the functional significance of the dysregulated genes using pathway analysis. For the upregulated genes, 20 significantly enriched pathways were identified for the differentially expressed genes between cilengitide-treated U87ΔEGFR cells and control cells (Table 1). For the downregulated genes, 7 significantly enriched pathways were identified (Table 2). Especially for the upregulated genes, the significantly enriched molecular pathways included apoptotic cleavage of cellular proteins, FasL/CD95L signaling, TNF receptor signaling pathway, and ceramide signaling pathway. Caspase 8, desmoplakin, and protein kinase C, zeta were included in these pathways and upregulated.

**Table 1:** **Significantly enriched pathways between cilengitide-treated U87ΔEGFR and control**

Pathwayname	Entrez gene ID	Gene symbol	P-value	Z score
NF-kB activation through FADD/RIP-1 pathway mediated by caspase-8 and -10	23586, 841	DDX58, CASP8	0.004	6.358
Organic cation/anion/zwitterion transport	55867, 6580	SLC22A11, SLC22A1	0.005	6.084
Cell adhesion molecules (CAMs)	1364, 4359, 941, :965	CLDN4, MPZ, CD80, CD58	0.014	3.213
NOSIP mediated eNOS trafficking	4846	NOS3	0.022	8.034
EPHB forward signaling	8867, 8997	SYNJ1, KALRN	0.026	3.585
Apoptotic cleavage of cellular proteins	1832, 841	DSP, CASP8	0.034	3.262
Regulation of RAC1 activity	2059, 8997	EPS8, KALRN	0.036	3.204
Activation, myristolyation of BID and translocation to mitochondria	841	CASP8	0.037	5.593
Fatty acids	1580	CYP4B1	0.037	2.177
Signaling in Immune system	23586, 3429, 6196, 6672, :841, 84433, 941	DDX58, IFI27, RPS6KA2, SP100, CASP8, CARD11, CD80	0.037	3.149
LKB1 signaling events	2011, 57521	MARK2,RPTOR	0.037	2.631
ErbB1 downstream signaling	2059, 4086, 5590	EPS8, SMAD1, PRKCZ	0.039	4.964
FasL/CD95L signaling	841	CASP8	0.044	4.964
NOSTRIN mediated eNOS trafficking	4846	NOS3	0.044	4.964
Organic anion transport	55867	SLC22A11,	0.044	4.964
Pyrimidine biosynthesis	7372	UMPS	0.044	4.964
Release of eIF4E	57521	PRTOR	0.044	4.964
Ceramide signaling pathway	5590, 841	PRKCZ, CASP8	0.046	2.899
TNF receptor signaling pathway	5590, 841	PRKCZ, CASP8	0.046	2.899
Thromboxane A2 receptor signaling	4846, 5590	NOS3, PRKCZ	0.048	2.853

**Table 2:** **Downregulated pathway**

Pathway name	Entrez gene ID	Gene symbol	P-value	Z score
Sphingolipid metabolism	8879	SGPL1	0.006	16.049
BH3-only proteins associate with and inactivate anti-apoptotic BCL-2 members	598	BCL2L1	0.015	8.496
G alpha (s) signalling events	134860, 4160	TAAR9, MC4R	0.022	3.717
Syndecan-3-mediated signaling events	4160	MC4R	0.032	5.52
Sphingosine 1-phosphate (S1P) pathway	8879	SGPL1	0.038	5.036
Signaling events mediated by the Hedgehog family	91653	BOC	0.042	4.771
Pentose phosphate pathway	8277	TKTL1	0.045	4.54

### Validation of the microarray results

**Figure 3:**
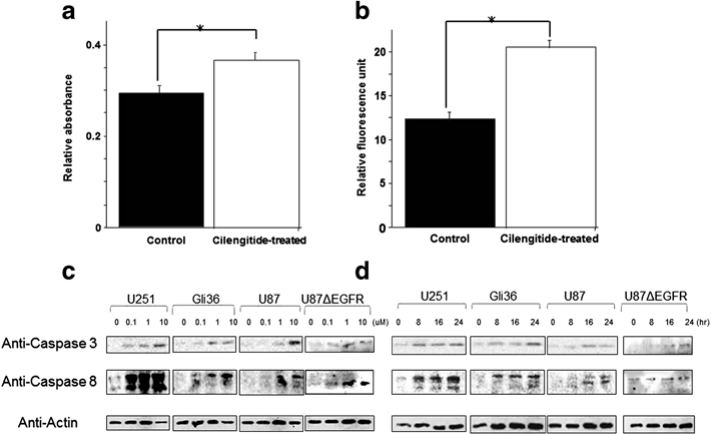
**QRT-PCR analysis and activation of caspase-3/7 and -8 in cilengitide-treated cells.** U87ΔEGFR cells were treated with 0.5 μM cilengitide for 16 h with the colorimetric protease assay kit. For the activity of caspase-8, the relative absorbance (RA) of U87ΔEGFR cell clusters were higher than control (0.27 ± 0.01 RA vs. 0.36 ± 0.01 RA, respectively; *P <* 0.05) (**a**). Cilengitide-induced apoptosis was also detected with the CellEvent™ Caspase-3/7 Green Detection Reagent. The RFU of U87ΔEGFR cell clusters were higher than control (35.4 ± 0.78 RFU vs. 16.5 ± 0.5 RFU, respectively; *P <* 0.05) (**b**). Immunoblot analysis revealed that caspases 3 and 8 were processed in all examined cell lines following treatment with cilengitide in a concentration- (**c**) and time-dependent manner (**d**).

To confirm the reliability of the results from the microarray analysis, caspase 8, protein kinase C, zeta, were verified by QRT-PCR analysis (Figure 3a, b). The relative expression of caspase 8 and protein kinase C, zeta in U87ΔEGFR cells incubated with cilengitide was significantly higher than those of the cells without cilengitide by 2.25-fold and 5.78-fold, respectively (P < 0.05).

### Capsase activation assay and caspase expression in western blotting

U87ΔEGFR cells were treated with 0.5 μM cilengitide for 16 h. Cilengitide-induced caspase-8 activity was detected with the colorimetric protease assay kit. The relative absorbance (RA) of U87ΔEGFR cell clusters were higher than control (0.27 ± 0.01 RA vs. 0.36 ± 0.01 RA, respectively; *P <* 0.05) (Figure 3a). U87ΔEGFR cells were loaded with 8 μM CellEvent™ Caspase-3/7 Green Detection Reagent then treated with the same concentration. Cilengitide-induced caspase 3/7 activity was detected with the CellEvent™ Caspase-3/7 Green Detection Reagent. The relative Fluorescence Unit (RFU) of U87ΔEGFR cell clusters were higher than control (35.4 ± 0.78 RFU vs. 16.5 ± 0.5 RFU, respectively; *P <* 0.05) (Figure 3b). The next series of experiments was designed to examine whether treatment with cilengitide induced caspase activation in other cell lines. Caspase activation was analyzed by using immunoblotting. Caspase 3 is produced as a 32-kDa proenzyme and cleaved into its 17 kDa active form. In U251, Gli36Δ5, U87MG, and U87ΔEGFR cells, cilengitide treatment induced the activated form of caspase 3. Caspase 8 is produced as a 55-kDa proenzyme and cleaved into its 30 kDa and 15 kDa active form. In U251, Gli36Δ5, U87MG, and U87ΔEGFR cells, cilengitide induced the activated form of caspase 9. Immunoblot analysis revealed that caspases 3 and 8 were processed in both cells in response to cilengitide in a concentration- (Figure 3c) and time-dependent manner (Figure 3d).

### Apoptosis analysis

**Figure 4:**
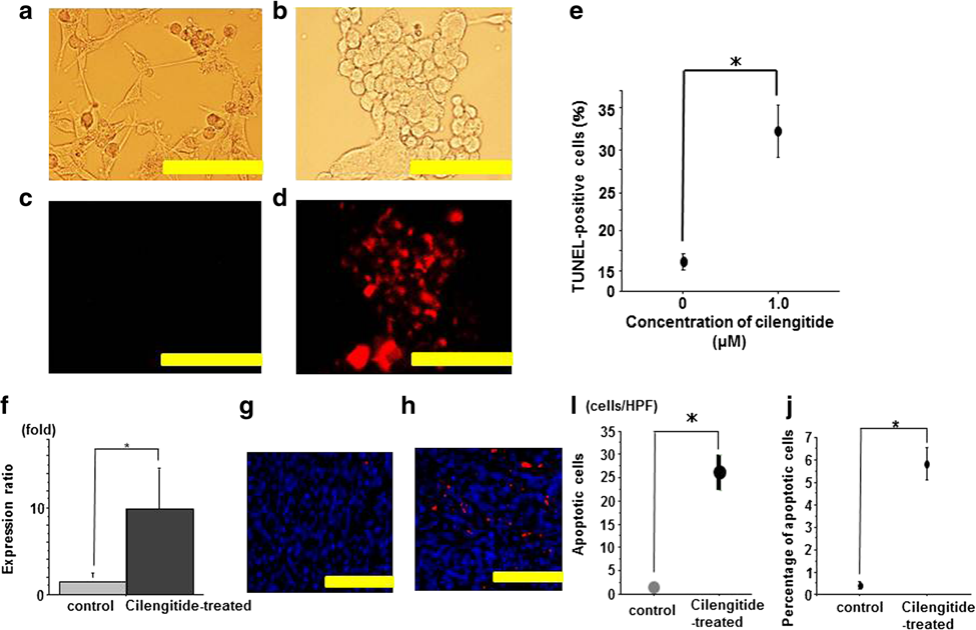
**Nuclear DNA fragmentation in cilengitide-treated cells and in U87ΔEGFR-derived xenografts.** To confirm the apoptosis of deformed glioma cells treated with cilengitide, the cells were stained with the In Situ Cell Death Detection Kit using TMR red. U87ΔEGFR cells in culture were composed of bipolar cells (**a**). They become spherical and agglutinated when cilengitide (1.0 μM) was added to their culture medium. Some of these deformed cells detached from the plate (**b**). Untreated U87ΔEGFR cells were negative (**c**), whereas U87ΔEGFR cell clusters treated with cilengitide were positive (**d**) (scale bar: 100 μm). The number of apoptotic cells in cilengitide-treated cells was significantly larger than in control. (*P < 0.05) (**e**). QRT-PCR analysis of cilengitide-treated caspase 8 gene expression in tumors treated with PBS or cilengitide. * *P* = .007 (**f**) (mean ± SE, n = 4) A subpopulation of apoptotic cells was visualized by TUNEL treatment (apoptotic cells: TMR red; nuclei: DAPI, blue) of U87**Δ**EGFR control xenografts (**g**) and U87**Δ**EGFR cilengitide-treated xenografts (**h**) (Scale bar = 100 μm). The control sections exhibited scattered red fluorescence, whereas more punctate red fluorescence was observed in the cilengitide-treated xenografts. To quantify the cytotoxic effect of cilengitide, the number or percentage of apoptotic cells per high-power field (HPF) in U87ΔEGFR control xenografts and U87ΔEGFR cilengitide-treated xenografts was assessed. The number (**i**) or percentage (**j**) of apoptotic cells in cilengitide-treated xenografts was significantly larger than in control xenografts (*P < 0.05) (mean ± SE, n = 5).

To confirm the apoptosis of the deformed glioma cells treated with cilengitide, the cells were stained with the In Situ Cell Death Detection Kit using TMR red. Originally, U87ΔEGFR cells in culture were composed of bipolar cells; however, they became spherical and agglutinated when cilengitide was added to the culture medium. Some of these deformed cells detached from the plate (Figure 4a, b, c, d). These detached U87ΔEGFR cells were not viable, as indicated by unsuccessful attempts of re-plating these cells in medium that did not contain cilengitide. U87ΔEGFR cell clusters were positive cells compared to control (35.4 ± 0.78 vs. 16.5 ± 0.5, respectively; *P <* 0.05) (Figure 4e).

### Cilengitide induces apoptosis in U87ΔEGFR-derived xenografts

The effect of cilengitide on the induction of apoptosis was examined in U87ΔEGFR-derived xenografts. At 5 days after implantation, the rats were administered cilengitide (1 mg/500 μL PBS) 3 times/week intraperitoneally, and the rats were killed at 18 days after implantation. Caspase 8 gene expression was analyzed with QRT-PCR and the induction of apoptosis in frozen sections of the U87ΔEGFR xenografts was examined under a fluorescent microscope. QRT-PCR revealed a statistically significant 10.1-fold increase in caspase 8 gene expression in Cilengitide treated tumors compared with control tumors (Figure 4f). A subpopulation of apoptotic cells were visualized by TUNEL treatment using the In Situ Cell Death Detection Kit (apoptotic cells: TMR red; nuclei: DAPI, blue). The control sections exhibited a smaller amount of red fluorescent cells (Figure 4g), whereas more scattered red fluorescent cells were observed in the cilengitide-treated xenografts (Figure 4h). To quantify the cytotoxic effect of cilengitide, the number of apoptotic cells per high-power field (HPF) in U87ΔEGFR control xenografts and U87ΔEGFR cilengitide-treated xenografts were assessed (Figure 4i). The number of apoptotic cells in U87ΔEGFR cilengitide-treated xenografts (26.2 ± 3.8 cells/HPF) was significantly higher than in U87ΔEGFR control xenografts (1.40 ± 0.6 cells/HPF) (P < 0.05). The average percentage of apoptotic cells was 5% in U87ΔEGFR cilengitide-treated xenografts (Figure 4j).

## Discussion

Cilengitide treatment induced morphological changes and cell detachment in glioma cells incubated in dishes and decreased cell viability in a dose and time-dependent manner. Microarray analysis showed that the expression of 265 genes was changed after cilengitide treatment. The expression of 214 genes was up-regulated 4-fold more and the expression of 51 genes was down-regulated to less than 25% of control and apoptotic signaling pathways were over-represented in the pathway analysis. In addition to the effect of cilengitide in cultured cells, cilengitide also induced apoptosis in U87ΔEGFR-derived xenografts, suggesting that the induction of apoptosis also occurs in vivo.

### Cytotoxic effect of Cilengitide

Cilengitide is an angiogenesis inhibitor that targets the integrins αvβ3 and αvβ5, which bind to ECM proteins such as vitronectin and fibronectin (Burke et al. 2002; Albert et al. 2006). Because integrins are expressed in tumor cells and tumor endothelial cells (Varner & Cheresh 1996a), it is speculated that cilengitide can inhibit tumor growth by at least 2 mechanisms: by targeting the tumor cells directly and by inhibiting tumor angiogenesis (Tucker 2003; Oliveira-Ferrer et al. 2008; Chatterjee et al. 2000). Previously, we described the anti-invasive effect of cilengitide as its direct effect on glioma cells (Onishi et al. 2012) And we also reported the multiple mechanism of cilengitide for malignant glioma (Kurozumi et al. 2012).

Recent studies have shown that various cells are dependent on integrin-mediated adhesion to specific ECM proteins for their growth and survival and that detachment induces a form of apoptotic cell death recognized as anoikis (Chatterjee et al. 2000; Hynes 2002; Oliveira-Ferrer et al. 2008; Alghisi et al. 2009). Other studies reported that cilengitide exerts direct cytotoxic effects on glioma cells via an as yet unknown mechanism (Mikkelsen et al. 2009; Maurer et al. 2009; Oliveira-Ferrer et al. 2008). In this study, we examined the mechanism of cilengitide-induced cytotoxicity in glioma cells.

### Microarray analysis

U87ΔEGFR cells were chosen for gene chip analysis because they have a more aggressive phenotype than other cell lines. ΔEGFR confers enhanced tumorigenicity on glioblastoma cells through elevated proliferation and reduced apoptotic rates *in vivo*. It will be important and interesting if we conduct a newly found cell death signaling pathway via integrin stimulation in this aggressive cell line.

Our data using the most sophisticated DNA microarray to date, profiling over 57000 genes, revealed the mechanism underlying the anti-glioma effect of cilengitide. After cilengitide treatment of glioma cells, apoptosis-related genes (i.e., caspase 8, desmoplakin, and protein kinase C, zeta) were upregulated. Apoptotic cleavage of cellular proteins, FasL/CD95L signaling, TNF receptor signaling pathway, and ceramide signaling pathway were included in the significantly enriched molecular pathways.

Apoptosis is regulated by a series of biochemical events that commit a cell to death. A common feature of cells undergoing apoptosis is the activation of caspases, a family of aspartic acid-directed proteases (Alnemri et al. 1996). Caspases are activated during apoptosis and cleave specific proteins, resulting in the irreversible commitment to cell death. The signal transduction proteins MEKK1, p21-activated kinase 2, and focal adhesion kinase are caspase substrates that contribute to the cell death response when cleaved.

FasL (CD95L) is a tumor necrosis factor (TNF)-related type II membrane protein (Suda et al. 1993). Fas (CD95) is a cell surface protein belonging to the TNF receptor superfamily, and is expressed in glioma cells (Husain et al. 1998). The binding of FasL to Fas induces the trimerization of Fas, and FADD (Fas associated via DD (death domain))/MORT1 binds to the trimerized FAS cytoplasmic region through the interaction of their respective DDs. Caspase-8 is then recruited to FADD/MORT1 through binding of the DED (dead effector domain) domains, which in turn may induce the self-activation of the protease domain (Nagata 1997).

TNF-R1-bound TRADD (TNF-receptor associated via DD) recruits FADD through DD interaction. In turn, FADD recruits procaspase-8 or -10, which are activated by proximity, via its DED. Protein kinase C, zeta, is also involved in the TNF receptor signaling pathway. Activation of caspase-8 and -10 is sufficient to initiate a signaling cascade that induces apoptosis (Schneider-Brachert et al. 2004).

Recently, similar changes in human umbilical vein endothelial cells (HUVECs) have been reported for S 36578-2, a novel RGD mimetic that selectively activates the αvβ3 and αvβ5 integrins(Maubant et al. 2006). This compound induces cell detachment and apoptosis by the direct activation of caspase-8. Aoudjit and Vuori (Aoudjit & Vuori 2001) reported that detachment-induced cell death in HUVECs resulted from the activation of the Fas pathway by FasL/Fas interaction, Fas-FADD complex formation, and caspase-8 activation. Previous reports on epithelial cells also documented the involvement of FADD and caspase-8 in detachment-induced apoptosis (Alghisi et al. 2009; Hynes 2002). Using human glioma cell lines expressing the αvβ3 and αvβ5 integrins, cilengitide caused a profound detachment and increase of apoptosis in glioma cells similar to what was observed in endothelial cells, suggesting that identical mechanisms might occur in both cell types (Oliveira-Ferrer et al. 2008).

### Clinical application of cilengitide for malignant glioma

There have been several reports on the preliminary results of phase I and II trials of cilengitide for recurrent or newly diagnosed malignant glioma. Cilengitide monotherapy or combination treatment with radiation and/or temozolomide is well tolerated and exhibits modest antitumor activity (Reardon et al. 2008a; Reardon et al. 2008b; Nabors et al. 2012). According to our results, in addition to the anti-angiogenic and anti-invasion effects of cilengitide(Kurozumi et al. 2012; Onishi et al. 2012), the cytotoxic effect of cilengitide was clearly shown. cilengitide inhibited integrin binding and activated caspase-8. This caspase-8 activation effect of cilengitide would enhance the effect of other cytotoxic therapies. Several preclinical studies have shown an enhanced antitumor effect of cilengitide when administered in combinatorial therapeutic regimens (Burke et al. 2002; Abdollahi et al. 2005; Tentori et al. 2008; Reardon et al. 2008a). Mikkelsen et al. demonstrated that cilengitide dramatically amplified the efficacy of radiation therapy in an animal glioma model (Mikkelsen et al. 2009). Kurozumi et al. demonstrated the enhanced therapeutic efficacy of an oncolytic virus on experimental glioma following pretreatment with cilengitide (Kurozumi et al. 2007).

## Conclusion

We showed the cytotoxic effect of cilengitide on glioma cells. Microarray analysis revealed the detailed mechanism of the cytotoxic effect of cilengitide. Cilengitide, an inhibitor of integrins, activated caspase-8 and induced apoptosis-related pathways.
